# Enhanced high β-carotene yeast cell production by *Rhodotorula paludigena* CM33 and in vitro digestibility in aquatic animals

**DOI:** 10.1038/s41598-024-59809-7

**Published:** 2024-04-22

**Authors:** Namphet Thumkasem, Thapanut On-mee, Chatchol Kongsinkaew, Supenya Chittapun, Soisuda Pornpukdeewattana, Mariena Ketudat-Cairns, Karun Thongprajukaew, Sompot Antimanon, Theppanya Charoenrat

**Affiliations:** 1 Department of Biotechnology, Faculty of Science and Technology, Thammasat University (Rangsit Center), Pathum Thani, 12120 Thailand; 2 Division of Fermentation Technology, School of Food Industry, King Mongkut’s Institute of Technology Ladkrabang, Bangkok, 10520 Thailand; 3 Center for Molecular Structure, Function, and Application, School of Biotechnology, Institute of Agricultural Technology, Suranaree University of Technology, Nakhon Ratchasima, 30000 Thailand; 4 Applied Aquatic Animal Nutrition Laboratory, Division of Health and Applied Sciences, Faculty of Science, Prince of Songkla University, Songkhla, 90110 Thailand; 5 Industrial Bioprocess Technology Research Team, Functional Ingredients and Food Innovation Research Group, National Center for Genetic Engineering and Biotechnology (BIOTEC), National Science and Technology Development Agency (NSTDA), Pathum Thani, 12120 Thailand

**Keywords:** Industrial microbiology, Applied microbiology, Animal biotechnology

## Abstract

This study assessed *Rhodotorula paludigena* CM33's growth and β-carotene production in a 22-L bioreactor for potential use as an aquatic animal feed supplement. Optimizing the feed medium's micronutrient concentration for high-cell-density fed-batch cultivation using glucose as the carbon source yielded biomass of 89.84 g/L and β-carotene concentration of 251.64 mg/L. Notably, using sucrose as the carbon source in feed medium outperforms glucose feeds, resulting in a β-carotene concentration of 285.00 mg/L with a similar biomass of 87.78 g/L. In the fed-batch fermentation using Sucrose Feed Medium, *R. paludigena* CM33 exhibited high biomass production rates (*Q*_*x*_) of 0.91 g/L.h and remarkable β-carotene production rates (*Q*_*p*_) of 2.97 mg/L.h. In vitro digestibility assays showed that *R. paludigena* CM33, especially when cultivated using sucrose, enhances protein digestibility affirming its suitability as an aquatic feed supplement. Furthermore, *R. paludigena* CM33's nutrient-rich profile and probiotic potential make it an attractive option for aquatic nutrition. This research highlights the importance of cost-effective carbon sources in large-scale β-carotene production for aquatic animal nutrition.

## Introduction

Carotenoids, essential tetraterpenoid pigments present in a range of organisms such as plants, fungi, protists, bacteria, yeasts, and archaea, play a crucial role in diverse biological processes. This includes exerting antioxidant activity and contributing to disease prevention, particularly in certain cancers and eye diseases^1,2^. Most animals, apart from a few arthropods, cannot synthesize these pigments de novo and must obtain them through their diet^3^, underscoring the importance of carotenoid research in biotechnological aspects.

*Rhodotorula paludigena*, has emerged as a distinguished bioresource for carotenoid production, outperforming other red yeasts and traditional carotenoid sources due to its unique qualities^4,5^. Renowned for its high β-carotene productivity, *R. paludigena* is adaptable to a variety of environmental conditions. It efficiently utilizes diverse carbon sources, a key advantage for industrial-scale production where cost-effectiveness and resource efficiency are crucial^5,6^. Its unique metabolic profile enables enhanced β-carotene production under specific conditions, a capability not commonly observed in other red yeast species. Furthermore, *Rhodotorula* yeast can be found in diverse environments such as water^7^, plants^8^, animals^9^, and human habitats^10^. Its ability to grow rapidly and complete production cycles can reduce operational space, water, and cost requirements, thereby minimizing environmental impact^11^. Additionally, its capacity to utilize low-cost agricultural and industrial wastes as carbon and nitrogen sources greatly contributes to waste reduction and environmental sustainability^6,12^. These attributes make *Rhodotorula* yeast an attractive candidate for applications across food, pharmaceutical, cosmetic, chemical, mariculture, and environmental protection industries^5,13^.

The strain *Rhodotorula paludigena* CM33, first isolated from castor seeds (*Ricinus communis*)^8^, has been identified for its significant lipid synthesis and carotenoid production capabilities^14^. Prior research by Sriphuttha et al.^5^ has extensively studied its effects on the growth, gene expression, intestinal microbial composition, disease resistance, and meat quality of whiteleg shrimp (*Litopenaeus vannamei*), demonstrating its potential as a probiotic supplement in shrimp feed. Despite these promising findings, high-cell-density fermentation for high β-carotene single cell protein production using *R. paludigena* CM33 remains unexplored. This process faces challenges from high production costs and nutritional imbalances in the fermentation medium^15^.

Along similar lines, Thumkasem et al.^4^ have extensively investigated the culture medium and cultivation conditions to regulate carotenoid production in *R. paludigena* CM33, employing the Response Surface Methodology using batch culture. The application of high-cell-density fermentation for high β-carotene yeast cell production with this strain remains unexplored. Balancing high cell density and carotenoid concentrations poses challenges due to carotenoid being non-growth associated in *R. paludigena* CM33.

To address these issues, fed-batch fermentation, a method that facilitated high-cell-density cultivation by progressively adding nutrients to the culture, was employed. This technique allowed for better control over substrate concentration, efficiently adjusting metabolism and mitigating the challenges posed by high substrate levels, which can lead to substrate inhibition and metabolic imbalances^16,17^. Despite its advantages, high-cell-density fermentation often encounters nutritional imbalances in the feed medium^18,19^. This was addressed in this research. This study focused on optimizing the fermentation process and feed medium to enhance β-carotene production by *R. paludigena* CM33 in a stirred tank bioreactor.

Although carbon sources play a crucial role in cell growth and productivity, they frequently represent a substantial portion of the total production costs in a standard fermentation process. In fact, carbon sources can account for over 80% of the total medium cost and more than 60% of the total production expenses^20^. This study used sucrose as an economical carbon source due to its affordability compared to glucose and its suitability for large-scale industrial processes. The enhancement of the titer, rate, and yield (TRY) in fermentation was focused on, particularly emphasizing volumetric productivity. This approach helps minimize both capital (bioreactor size) and operating (fermentation duration) costs, aligning with our primary objective of optimizing carotenoid production while reducing overall expenses.

Building upon the established potential of *R. paludigena* CM33 as a probiotic supplement for shrimp, as demonstrated by Sriphuttha et al.^5^, this study aimed to further explore the utility of this strain in aquaculture. Therefore, this research focused on harnessing high β-carotene yeast cell produced by *R. paludigena* CM33, investigating its suitability as a dietary supplement to enhance the growth and/or health of aquatic animals. A key aspect of this investigation was the evaluation of the in vitro digestibility of protein (IVDP) and in vitro digestibility of carbohydrate (IVDC) based on digestive enzyme extracts from aquatic animals, including Nile tilapia (*Oreochromis niloticus*), striped catfish (*Pangasianodon hypophthalmus*), and whiteleg shrimp (*L. vannamei*), to determine its suitability as a feed supplement to promote growth of aquatic animals.

## Materials and methods

### Microorganism and inoculation preparation

The yeast strain, *Rhodotorula paludigena* CM33, obtained from Suranaree University of Technology, Thailand^8^ was cultured on YPD agar plates, comprising Yeast extract (10 g/L), Peptone (10 g/L), Dextrose (20 g/L), and agar (20 g/L). These plates were incubated at 30 °C for 48 h, following which they were stored at 4 °C. The primary inoculum was prepared by transferring a 48-h *R. paludigena* CM33 colony on an agar plate to a 250-mL Erlenmeyer flask containing 40 mL of YPD medium. After 24 h of incubation at 30 °C and 250 rpm, the optical density of the primary inoculum at 600 nm (OD_600_) was adjusted to 12.0 by dilute with the medium.

The secondary inoculum was prepared by transferring 10 mL of the primary inoculum to a 500-mL flask with 90 mL of Thumkasem-2023 medium. This medium was adapted from Miao et al.^12^ and Thumkasem et al.^4^, consisted of glucose (20 g/L for flask culture and 40 g/L for bioreactor culture), KH_2_PO_4_ (7 g/L), yeast extract (0.5 g/L), Na_2_HPO_4_ (2.5 g/L), (NH_4_)_2_SO_4_ (6.2 g/L), MgSO_4_·7H_2_O (1.5 g/L), FeCl_3_ (0.075 g/L), CaCl_2_·2H_2_O (0.199 g/L), and ZnSO_4_·7H_2_O (0.02 g/L), with the pH adjusted to 6.0. The secondary inoculum was also incubated at 30 °C and 250 rpm for 24 h. As previously, the OD_600_ was adjusted to 12.0 before use as an inoculum for the bioreactor.

### Fed-batch fermentation of *Rhodotorula paludigena* CM33

A 22-L stirred-tank bioreactor (Biostat C Plus, Sartorius Stedim Biotech, Germany) with 4.75 L of Thumkasem-2023 medium was employed, following the method of Thumkasem et al.^4^. The secondary inoculum of 250 mL was introduced to achieve an initial OD_600_ of approximately 0.6. The fermentation conditions included a temperature of 30 °C, agitation at 700 rpm (83 mm impeller diameter and 3.04 m/s tip speed), aeration at 1.0 vvm, and pH maintained at 6.0 using 5.0 M NaOH and 5.0 M H_3_PO_4_. The batch fermentation ended when the dissolved oxygen tension (DOT) rapidly increased, signaling glucose depletion, typically around 20 h. Subsequently, the fed-batch fermentation with DOT stat feed control began based on the strategy from Kongsinkaew et al.^21^. The agitation speed was increased to 900 rpm for the duration of the 76-h fed-batch process.

The addition of feed medium was regulated to maintain DOT levels within 30–70% air saturation during *Rhodotorula* yeast fermentation, a critical step to enhance β-carotene production as it is a non-growth associated process in *Rhodotorula* yeast^22^. This control strategy ensures optimal conditions for both growth and non-growth associated product synthesis, facilitating complete substrate consumption before each feed cycle, favoring β-carotene production by reducing growth rates and substrate concentrations^22^. Substrate depletion triggering DOT levels above 70% activates the medium feed pump, while levels below 30% deactivate it to prevent oxygen limitation. This approach effectively balances oxygen availability for growth and biosynthesis, promoting β-carotene production by minimizing growth rates and substrate concentrations.

### Effect of micronutrient concentration in Feed Media composition on high cell density fermentation

The optimum culture medium for batch fermentation, Thumkasem-2023 medium, was derived from Thumkasem et al.^4^. For fed-batch fermentation, the macronutrients, both carbon source and nitrogen source, were increased to achieve high cell density. However, simply raising the macronutrient concentration is not sufficient to reach the goal; hence, micronutrient increase is also vital, as the trace element concentration in feed medium is often increased in fed-batch high-cell-density cultivation of *Pichia pastoris*^21^. Therefore, four feed medium formulas with variations in micronutrient concentration and type of carbon source were designed, as shown in Table 1.

**Table 1:** Component concentration in each formula of feed medium.

Components	Concentration of components (g/L)			
	Feed Medium 1	Feed Medium 2	Feed Medium 3	Sucrose Feed Medium
Carbon source				
Glucose	500	500	500	–
Sucrose	–	–	–	500
Nitrogen source				
(NH_4_)_2_SO_4_	77.5	77.5	77.5	77.5
Micronutrients				
KH_2_PO_4_	7	14	21	21
Yeast extract	0.5	1.0	1.5	1.5
Na_2_HPO_4_	2.5	5.0	7.5	7.5
MgSO_4_·7H_2_O	1.5	3.0	4.5	4.5
FeCl_3_	0.075	0.150	0.225	0.225
CaCl_2_·2H_2_O	0.199	0.398	0.597	0.597
ZnSO_4_·7H_2_O	0.02	0.04	0.06	0.06

The concentrations of the carbon source and nitrogen source were set at an optimal mass ratio of 6.45:1.0, equating to 500 g/L for either glucose or sucrose and 77.5 g/L for (NH_4_)_2_SO_4_ Thumkasem et al.^4^. The micronutrients had their concentrations varied to 1, 2, and 3 times the optimal concentration specified for batch fermentation, as reported by Thumkasem et al.^4^ (Table 1). The determined optimal micronutrient concentration was then incorporated into the feed medium that used sucrose as the carbon source, termed the Sucrose Feed Medium (Table 1). The high-cell-density fed-batch fermentation of *R. paludigena* CM33 was run as described in the previous section. During cultivation, approximately 30–40 mL of culture were sampled periodically to be analyzed for cell growth, substrate consumption, ammonium sulfate utilization, and β-carotene production.

### In vitro digestibility for feed additive feasibility

#### Animal collection and extraction of digestive enzymes

Economically important aquatic animals were used as a source of enzymes for determining in vitro digestibility. The Institutional Animal Care and Use Committee at Prince of Songkla University, Songkhla, Thailand approved all animal protocols (Code 2022-Sci11-018), including preparation, sampling, collection, and euthanasia, for specimens collected from private farms in Songkhla province, Thailand. These animal protocols adhered to the procedures prescribed by the Institute for Animals for Scientific Purposes Development (IAD), National Research Council of Thailand (License No. U1-06514-2560, date of expiry October 22, 2027), which were conducted in compliance with the ARRIVE guidelines. The intestines from juvenile Nile tilapia (9.76–13.7 g body weight), striped catfish (2.35–3.15 g body weight), and whiteleg shrimp (1.02–1.62 g body weight) were removed on ice, mixed with 0.2 M Na_2_HPO_4_-NaH_2_PO_4_ pH 8.2 (1:3 w/v ratio), and then homogenized for 20 s using a tissue micro‐homogenizer (THP‐220; Omni International, GA, USA). The supernatant was collected after centrifugation at 15,000×*g* for 30 min at 4 °C and then dialyzed overnight against 50 mM Na_2_HPO_4_-NaH_2_PO_4_ pH 8.2. The dialyzed crude enzymes were kept at − 20 °C until used to test in vitro digestibility.

#### Substrate preparation for in vitro digestibility assay

For the in vitro digestibility assay, three yeast substrates were prepared and used as comparative (control) substates. The first, named SB, was *Saccharomyces boulardii* that had been cultivated in a 5-L bioreactor using batch fermentation, following the conditions set by Thumkasem et al.^4^. The second, referred to as CSC, a commercial source of *Saccharomyces cerevisiae* (≥ 2 × 10^10^ cells/g) purchased from Star Yeast 370, ICC Brazil Pet, São Paulo, SP, Brazil. The third, termed CSCB, was a commercial blend containing *Saccharomyces cerevisiae*, enhanced with multi-strain probiotics such as *Lactobacillus cerevisiae*, *Bacillus subtilis*, and *Lactobacillus acidophilus*, and further fortified with vitamins, minerals, proteins, and amino acids. The CSCB, which contained over 10^8^ cells/g, was purchased from SF. Farm, Samut Sakhon, Thailand. All three substrates underwent freeze-drying using the Delta 2–24 LSC equipment from Martin Christ Gefriertrocknungsanlagen GmbH in Germany and were sieved before performing in vitro digestibility.

For a comprehensive analysis, these three treatments (SB, CSC, and CSCB) were compared against three other substrates: RPO, which was *R. paludigena* CM33 cultured in a 22-L bioreactor using batch fermentation^4^; RPG, which was the same strain but grown in a 22-L bioreactor using fed-batch fermentation and fed with Feed Medium 3; and RPS, where it was cultured similarly in a 22-L bioreactor but fed with Sucrose Feed Medium.

#### Determination of in vitro digestibility

To assess in vitro digestibility, enzymes extracted from aquatic animals were employed, with yeast specimens serving as substrates following the methodology of Thongprajukaew et al.^23^. The reaction mixtures were prepared, comprising 5 mg of dried yeast, 10 mL of 50 mM Na_2_HPO_4_-NaH_2_PO_4_ pH 8.2, 50 µL of 5 g/L chloramphenicol, and 125 µL of the dialyzed crude enzyme extract. The mixtures were incubated at 25 °C for 24 h. The enzyme reaction was terminated by heating at 100 °C for 10 min.

For protein digestibility, 200 µL of the digested solution was combined with 2 mL of 50 mM Na_2_HPO_4_-NaH_2_PO_4_ pH 8.2 and 1 mL of 1 g/L trinitrobenzene sulfonic acid. This mixture was then incubated at 60 °C in the dark for one hour, then the reaction was stopped by adding 1 mL of 1 M HCl. The protein digestibility was spectrophotometrically determined by measuring the increase in liberated reactive amino groups of cleaved peptides at 420 nm against the linear range of *DL*-alanine. The values are expressed as mmol of *DL*-alanine equivalent per gram of sample.

For assessing carbohydrate digestibility, 500 µL of the digested sample was blended with 250 µL of 10 g/L dinitrosalicylic acid and heated to 100 °C for 5 min. The increase in released sugars was measured at 540 nm, using maltose's linear range as a reference, and expressed as mmol of maltose per gram of sample.

### Analytical methods

#### Biomass determination

To determine biomass, 1.5 mL of culture broth was centrifuged at 7378×*g* for 5 min. The obtained cell pellet was washed using 0.2 M H_3_PO_4_ and then with distilled water before drying at 80 °C until consistent weight. The dry cell pellet's weight was measured and expressed as grams per liter^24^.

#### Determination of reducing sugar

The modified 3,5-dinitrosalicylic acid (DNS) method, as described by Miller^25^, was applied for quantifying reducing sugars. After appropriate dilution of the sample, 0.5 mL of sample was mixed with 0.5 mL of DNS reagent and boiled for 10 min. This was cooled rapidly and diluted with 10 mL of distilled water before measuring its absorbance at 540 nm using a spectrophotometer (Metertech SP880, Taiwan). The residual glucose concentration was calculated by referring to a glucose standard curve.

Sucrose concentration was analyzed using the modified method of Laopaiboon et al.^26^. The samples were digested with a 0.2 M HCl solution in a 1:1 sample-to-HCl solution volume ratio, shaken well, boiled for 20 min, and allowed to cool. Then, the mixture was neutralized by a NaOH solution. The concentration of the sugars was then determined using the modified DNS method, referencing a glucose standard curve, and reported as a glucose equivalent.

#### Determination of ammonium sulfate

To determine ammonium sulfate concentration, the modified Phenol-Hypochlorite method, adapted from Weatherburn^27^, was utilized. The procedure involved the addition of 5 mL of Phenol-Nitroprusside solution to the 1 mL of sample, followed by the addition of 5 mL of Alkaline hypochlorite solution. After incubating the mixture at 37 °C for 20 min, absorbance was measured at 625 nm using a spectrophotometer (Metertech SP880, Taiwan) and compared against an ammonium sulfate standard curve.

#### Carotenoids extraction and quantification

The method from Thumkasem et al.^4^ was used for carotenoid extraction. The fermented broth (1.5 mL) was centrifuged at 7378×*g* for 5 min, and the resulting cell pellet was washed with distilled water and dried in a freeze-dryer (Gold-sim, Miami, USA). Carotenoids were then extracted using 850 µL of Dimethyl sulfoxide (DMSO) and 600 µL of acetone. The clear crude extract solution was recovered after centrifuging at 7378×*g* for 5 min, and β-carotene was quantified using High-Performance Liquid Chromatography (HPLC) (1260 Infinity II, Agilent Technology, Santa Clara, USA) using a modified method of Khumrangsee et al.^28^. The HPLC analysis was performed using a C18 reverse-phase column (ZORBAX Eclipse Plus, 150 mm × 4.6 mm × 5 µm) and a diode array detector at a wavelength of 450 nm. The mobile phase consisted of acetonitrile, dichloromethane, and methanol (7:2:1, v/v/v) at a flow rate of 1 mL/minute. The carotenoid concentration was evaluated using a calibration curve of a β-carotene standard curve (Sigma-Aldrich, USA).

#### Nutritional composition analysis

Yeast cell nutritional composition was examined at the Institute of Food Research and Product Development Food Quality Assurance Service Center, Chulalongkorn University, Thailand. The analysis utilized multiple methods from the AOAC (2016), specifically: 934.01 (Moisture), 2001.11 (Crude protein), 920.39 (Crude lipid), 978.10 (Crude fiber), 942.05 (Ash), 927.02 (Calcium), and 965.17 (Phosphorus). Nitrogen-free extract was calculated by 100 – (moisture + crude protein + crude lipid + crude fiber + ash). All values are expressed on a dry matter basis.

#### Kinetic parameter calculation

The essential kinetic parameters for evaluating the growth and β-carotene production of *R. paludigena* CM33 were determined, namely specific growth rate ($\mu$), biomass yield based on the substrate consumed (*Y*_*x/s*_), product yield based on the substrate consumed (*Y*_*p/s*_), yield coefficient based on the biomass (*Y*_*p/x*_), yield coefficient of product on a nitrogen substrate (*Y*_*x/N*_), yield coefficient of biomass on a nitrogen substrate (*Y*_*p/N*_), volumetric productivities of biomass during a specific period (*Q*_*x*_), and β-carotene productivity that occurs during a specific period (*Q*_*p*_). These parameters were computed using the equations as outlined by Malisorn et al.^29^.

#### Statistical analysis

All values represent the mean of three replications and are presented with their standard deviation (mean ± SD). The fed-batch cultivation data were analyzed using IBM SPSS version 29.0. The comparison of means was conducted using Tukey's HSD test, with a 95% confidence level (P < 0.05). To assess the nutritional compositions of *R. paludigena* CM33 grown on varying carbon sources, the nutrient data from fed-batch fermentations using glucose and sucrose were compared. These comparisons were conducted using the Student t-test, with significance levels denoted as P < 0.05 (*), P < 0.005 (**), and P < 0.001 (***).

## Results and discussion

### Impact of micronutrient concentration in Feed Medium on *R. paludigena* CM33 high cell density fed-batch fermentation

The effectiveness of a growth-promoting and β-carotene-producing Feed Medium for fed-batch fermentation was explored further in this research, using an optimal medium for batch fermentation of *R. paludigena* CM33^4^. In feed medium, the carbon and nitrogen source concentrations were elevated to 500 g/L and 77.2 g/L, respectively. Optimization of micronutrient concentrations to enhance cell density and β-carotene content, in sync with the elevated C-source and N-source, was the focus.

**Figure 1:**
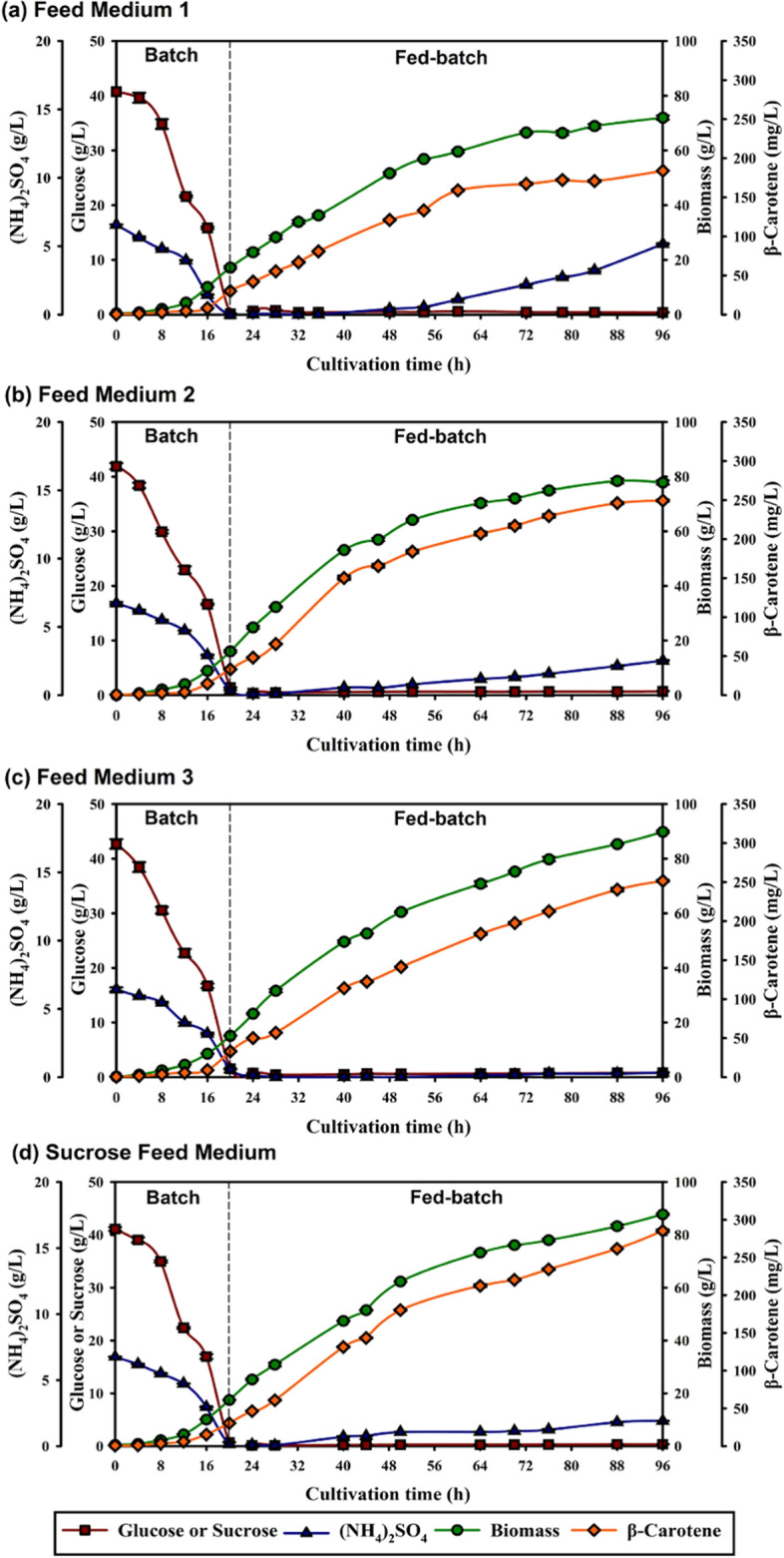
Comparison of fed-batch fermentation profiles of *R. paludigena* CM33 in the 22L-bioreactor using different feed media (**a**) Feed Medium 1, (**b**) Feed Medium 2, (**c**) Feed Medium 3, and (**d**) Sucrose Feed Medium. Feed medium 1, 2, and 3 used glucose as a carbon source with a concentration of micronutrients of 1, 2, and 3 times, respectively, compared to the optimum concentration for batch fermentation. Sucrose Feed Medium uses sucrose as a carbon source with a concentration of micronutrients 3 times higher than the optimum concentration for batch fermentation.

Fed-batch fermentation was executed in a 22-L bioreactor, starting with batch fermentation using optimal cultivation conditions and medium^4^. In the batch cultivation phase, glucose and ammonium sulfate were exhausted in 20 h (Fig. 1), indicating the cell's response to the cultivation system. At this point, the average biomass reached 16.63 ± 1.02 g/L, and the average β-carotene concentration was 32.28 ± 1.63 mg/L for all experiments (Fig. 1a–d). Subsequently, the fed-batch cultivation phase began by replenishing the Feed Medium with varying concentrations of micronutrients, as described in Materials and Methods and Table 1. The fed-batch cultivation phase was run for 76 h using DOT stat feed control (total cultivation time of 96 h), leading to different biomass, β-carotene, yields, and productivities for each feed medium, as indicated in Table 2.

**Table 2:** Comparison of the biomass and β-carotene concentrations of *R. paludigena* CM33 with different feed media in a 22-L bioreactor at 96 h.

Parameters	Feed Medium 1	Feed Medium 2	Feed Medium 3	Sucrose Feed Medium
*µ* (h^−1^)	0.024 ± 0.00^b^	0.027 ± 0.00^a^	0.029 ± 0.00^a^	0.028 ± 0.00^a^
Final biomass (g/L)	72.00 ± 0.75^d^	77.89 ± 0.77^c^	89.84 ± 0.41^a^	87.78 ± 0.30^b^
Total biomass (g)	538.42 ± 5.66^c^	616.88 ± 6.12^b^	707.97 ± 3.21^a^	709.24 ± 2.43^a^
Final β-carotene (mg/L)	184.13 ± 0.00^c^	249.42 ± 1.33^b^	251.64 ± 1.06^b^	285.00 ± 1.84^a^
Total β-carotene (mg)	1376.90 ± 0.00^c^	1975.38 ± 30.54^b^	1982.93 ± 8.38^b^	2036.01 ± 14.88^a^
*Y*_*x/s*_ (g cell/g glucose)	0.36 ± 0.00^c^	0.37 ± 0.00^b^	0.42 ± 0.00^a^	0.37 ± 0.00^b^
*Y*_*p/s*_ (mg β-carotene/g glucose)	0.91 ± 0.00^c^	1.20 ± 0.01^ab^	1.18 ± 0.01^b^	1.22 ± 0.01^a^
*Y*_*p/x*_ (mg β-carotene/g cell)	2.56 ± 0.03^c^	3.20 ± 0.06^a^	2.80 ± 0.02^b^	3.25 ± 0.03^a^
*Y*_*x/N*_ (g cell/g nitrogen)	2.30 ± 0.02^d^	2.41 ± 0.02^c^	2.71 ± 0.01^a^	2.56 ± 0.01^b^
*Y*_*p/N*_ (mg β-carotene/g nitrogen)	5.89 ± 0.00^c^	7.72 ± 0.12^b^	7.60 ± 0.03^b^	8.31 ± 0.05^a^
*Q*_*x*_ (g cell/L·h)	0.75 ± 0.01^d^	0.81 ± 0.01^c^	0.94 ± 0.00^a^	0.91 ± 0.01^b^
*Q*_*p*_ (mg β-carotene/L·h)	1.92 ± 0.00^c^	2.59 ± 0.04^b^	2.62 ± 0.01^b^	2.97 ± 0.02^a^

Data are given as means ± SD (*n* = 3). Means with different superscript letters within the same row were significantly different (P < 0.05).

After 96 h of fed-batch cultivation, it became evident that Feed Medium 1, characterized by the lowest concentration of micronutrients, resulted in the least favorable outcomes in terms of biomass and β-carotene production. The biomass and β-carotene levels were 72.00 ± 0.75 g/L and 184.13 ± 0.00 mg/L, respectively, as shown in Table 2. This low level of cell proliferation and growth can be attributed to potential constraints arising from the limited availability of some components in micronutrients^19^. As observed in Fig. 1a, there was a continuous accumulation of ammonium sulfate during the fed-batch cultivation with Feed Medium 1 until the end of the process, with the ammonium sulfate concentration reaching 5.17 ± 0.04 g/L at 96 h. The accumulation of ammonium sulfate might have occurred from the cultivation limitation under conditions of low micronutrient availability, causing the microorganism to experience nutrient imbalance^18^.

To address the challenges posed by micronutrient limitations, the concentration of micronutrients was doubled in Feed Medium 2. This adjustment yielded notably improved results, with biomass and β-carotene concentrations reaching 77.89 ± 0.77 g/L and 249.42 ± 1.33 mg/L, respectively. These findings indicate that increasing the concentration of micronutrients has an impact on nutrient balance, consequently boosting biomass and β-carotene output. Additionally, in pursuit of higher β-carotene yields, a state of nitrogen limitation was intentionally introduced, as supported by Cescut et al.^30^ and Saenge et al.^31^.

Although the increased in micronutrient concentration improved outcomes, an accumulation of ammonium sulfate reaching a concentration of 2.54 ± 0.03 g/L at 96 h, as shown in Fig. 1b. This persistent ammonium sulfate accumulation suggests that while the adjustment addressed certain nutrient imbalances, additional factors involving co-factors or micronutrients may require further investigation. It indicates that the mere presence of carbon and nitrogen sources is insufficient if specific co-factors or balanced nutrition is lacking. This deficiency can prevent the yeast *R. paludigena* CM33's ability to effectively utilize these resources, impacting cell growth, metabolic processes, and biosynthesis, as noted in Li et al.^18^.

In both, the enhanced micronutrient concentration and nitrogen limitation scenarios, the importance of cultivation conditions and nutrient balance is underscored. These factors are crucial in determining the performance of *R. paludigena* CM33 in terms of biomass and β-carotene production. The findings highlight the intricacies involved in bioprocess optimization and the need for a finely tuned nutrient equilibrium to maximize yields in biotechnological applications. Moreover, the improvements in growth parameters and carotenoid production, detailed in Table 2, further reinforce these conclusions.

Despite the similar initial concentrations of ammonium sulfate across all feed media, in Feed Medium 3 a complete depletion of ammonium sulfate was observed, indicating a balanced utilization rate with glucose. It yielded the highest biomass and β-carotene concentrations (89.84 ± 0.41 g/L and 251.64 ± 1.06 mg/L, respectively) when compared to Feed Medium 1 and 2. While the yeast extract provided additional nitrogenous compounds, its higher concentration in Feed Medium 3 (1.5 g/L) did not necessarily translate to increased nitrogen availability for growth, as evidenced by the complete depletion of ammonium sulfate which is 51.67 times higher concentration to yeast extract. This observation implies a potential shift in the yeast's metabolic focus from growth to secondary metabolite production, such as carotenoids, under conditions where nitrogen sources were exhausted (Fig. 1c), aligning with the findings of Cescut et al.^30^ and Saenge et al.^31^. This phenomenon of metabolic shift, rather than straightforward nitrogen limitation, may contributed to the enhanced carotenoid accumulation observed in Feed Medium 3. Since the batch phase was consistent across all experiments, regardless of the feed medium used, the observed differences in each experiment can primarily be attributed to variations in the micronutrient concentration. This in-depth analysis of the effect of micronutrients shows the potential of Feed Medium 3 for further development in larger scale processes, notably for industrial applications.

In the *Rhodotorula* yeast, the nitrogen limitation led to the decreasing in growth rate and carotenoid accumulation through residual carbon source utilization. Changes in carbon source to citrate increased citrate accumulation, enhancing carotenoid synthesis^32,33^. The inclusion of inorganic salts, specifically disodium hydrogen phosphate (Na_2_HPO_4_) and potassium dihydrogen phosphate (KH_2_PO_4_), serves a dual purpose in the culture medium. These salts function as buffers, maintaining the medium's pH, which is crucial for optimal cellular pH balance during the growth and carotenoid synthesis. This role of pH in influencing both growth and carotenoid production in *Rhodotorula* yeast has been reported by Aksu et al.^34^ and Kot et al.^35^. Their research underscores the significant impact that pH has on the overall efficacy of the bioprocess, particularly in terms of both yeast growth and carotenoid yield. Increasing the concentration of magnesium ions can have a significant impact on both growth and carotenoid synthesis^36^, as magnesium ions act as essential cofactors in many cellular processes^37^. Elevated levels of magnesium boost the synthesis of carotenoids by stimulating the function of acetyl CoA carboxylase^38^, while the lack or excessive quantity of magnesium results in metabolic changes^39^. This might potentially have an adverse impact on the process of carotenoid production.

### Comparison of the effect of Feed Medium with glucose and sucrose on cell growth and β-carotene production

This study evaluates the use of sucrose as an alternative carbon source to glucose in the fed-batch cultivation of *R. paludigena* CM33, primarily to reduce production costs. The data presented in Table 2 demonstrates that substituting glucose with sucrose in the feed medium resulted in comparable biomass production (87.78 ± 0.30 g/L) but significantly enhanced β-carotene levels (285.00 ± 1.84 mg/L). The increase in β-carotene production during the fed-batch phase, when transitioning from glucose to sucrose, can be attributed to a metabolic shift in *R. paludigena* CM33, as evidenced by research from Lee et al.^40^ and Sánchez et al.^41^. This shift involves the enzymatic breakdown of sucrose into glucose and fructose, a process catalyzed by invertase,detailed in studies by Park et al.^42^ and Gong et al.^43^. The presence of both glucose and fructose alters the yeast’s metabolic flux, with fructose entering glycolysis at a point that bypasses the regulatory phosphofructokinase step. This variation allows for a more efficient flow through the pentose phosphate pathway, essential for producing NADPH, a vital cofactor in carotenoid biosynthesis. The dual availability of these monosaccharides enhances metabolic efficiency and NADPH availability, crucial for the increased synthesis of β-carotene. This phenomenon emphasizes the importance of substrate diversity in regulating metabolic pathways and enhancing secondary metabolite production in yeasts^43^.

In the Sucrose Feed Medium, an unexpected accumulation of ammonium sulfate was noted, reaching 1.92 ± 0.01 g/L at 96 h (Fig. 1d). This accumulation, contrasting with the glucose feed medium, may indicate a differential carbon utilization strategy by *R. paludigena* CM33. Notably, despite this availability of ammonium sulfate, β-carotene accumulation in the sucrose medium was the highest among the tested media by the experiment's end.

This finding calls for a reassessment of the previously held assumption, as mentioned in Cescut et al.^30^ and Saenge et al.^31^, which proposed that low levels of ammonium sulfate were critical for boosting carotene production. It now appears that the link between ammonium sulfate concentration and β-carotene synthesis is more intricate than originally thought. This complexity might be attributed to changes in metabolic pathways induced by sucrose, potentially channeling the metabolic flux towards an enhanced β-carotene synthesis, as previously discussed^43^. Furthermore, it is important to consider that β-carotene is synthesized as a secondary metabolite^22^. Therefore, the growth rate of the yeast is another crucial factor that must be taken into account. This factor could significantly influence the overall metabolic activity and hence the production of β-carotene, underscoring the multifaceted nature of this bioprocess.

Interestingly, the metabolic shift induced by using sucrose instead of glucose initially led to a temporary slowdown in microbial growth. However, this was accompanied by an increase in β-carotene production, potentially as a response to cellular stress^44^. The underlying biochemical process involves sucrose and glucose enhancing the synthesis of acetyl coenzyme A (CoA), a precursor for mevalonic acid, which is a key substrate in carotenoid production^45^. Kilian et al.^46^ reported that disaccharides such as sucrose, maltose, and cellobiose can significantly boost carotenoid production up to 12-fold, while high glucose concentrations might inhibit it.

Considering the titer, rate, and yield (TRY) metrics, the sucrose feeding system showed 13.26%, 13.36%, and 5.70% higher β-carotene concentration, volumetric productivity, and yield based on substrate consumed as glucose equivalent compared to the glucose feeding system, respectively. Additionally, using sucrose as the carbon source in the feed medium presents significant benefits over conventional glucose-based media, particularly for enhancing β-carotene production and reducing costs. One of the key advantages of using sucrose is its cost-effectiveness, as it is more than twice as affordable compared to glucose. This economic benefit, combined with its effectiveness in boosting β-carotene yields, makes sucrose an attractive alternative for feed media in fermentation processes. In the context of large-scale industrial production of microbial carotenoids, the selection of low-cost carbon sources is crucial. These findings hold significant implications for the commercial and industrial bioprocessing sectors, suggesting that sucrose is a viable alternative for the development and optimization of bioprocesses for protein and carotenoid production.

### Comparative analysis of β-carotene production techniques

Table 3 presents a comparative analysis with other literature reviews focusing on fed-batch cultivation^47–52^. Among these, *R. paludigena* CM33 stands out for its superior β-carotene production, particularly notable when employing a DOT stat feed control strategy. This strain outperformed others, such as *S. roseus*^51^ and *R. mucilaginosa*^48^, in terms of biomass yields and β-carotene production. Specifically, it achieved biomass yields of 89.84 ± 0.41 g/L with glucose and 87.78 ± 0.30 g/L with sucrose, and β-carotene yields of 2.80 ± 0.02 mg/g cell with glucose and 3.25 ± 0.03 mg/g cell with sucrose. The productivity rates were also remarkable higher than that reported in all literature reviewed^47–52^ in Table 3. The biomass production rates (*Q*_x_) of 0.94 ± 0.00 g/L.h for glucose and 0.91 ± 0.01 g/L.h for sucrose, and β-carotene production rates (*Q*_*p*_) of 2.62 ± 0.01 mg/L.h for glucose and 2.97 ± 0.02 mg/L.h for sucrose were achived in this present work.

**Table 3:** Comparison of the performance of fed-batch culture conditions for biomass, β-carotene production, and productivity in red yeast.

Microorganisms	Carbon sources	Culture conditions	Biomass (g/L)	β-Carotene (mg/g cell)	*Q*_*x*_ (g/L·h)	*Q*_*p*_ (mg/L· h)	References
*Sporobolomyces roseus* CFGU-S005	Pasta processing waste hydrolysate	Stirred tank bioreactor at 25 °C, 150 rpm, pH = 5.0 in 120 h	12.8	0.27	0.11	0.03	Villegas-Méndez et al.^47^
*Rhodotorula mucilaginosa* CCT 7688	Sugar cane molasses	Incubated in a shake flask at 25 °C, 180 rpm, pH = 6.0 in 216 h	16	0.23	0.07	0.02	Dias Rodrigues et al.^48^
*Sporidiobolus salmonicolor* CBS 2636	Glycerol	Stirred tank bioreactor at 25 °C, 180 rpm, pH = 4.0 in 96 h	5.4	0.82	0.06	0.05	Colet et al.^49^
*Rhodotorula glutinis*	Crude glycerol	Airlift bioreactor at 24 °C, pH = 5.5, < 300 rpm in 120 h	46.4	1.417	0.39	0.55	Yen et al.^50^
*Sporobolomyces roseus*	Spent coffee grounds , sugars	Stirred tank bioreactor at 28 °C, pH = 5.5 in 65 h	36.8	0.42	0.57	0.24	Petrik et al.^51^
*Rhodotorula glutinis* mutant 32	Sugar cane molasses	Stirred tank bioreactor at 28 °C, 500 rpm, pH = 6.0 in 96 h	78	2.03	0.81	1.65	Bhosale and Gadre^52^
*Rhodotorula paludigena* CM33	Glucose	Stirred tank bioreactor at 30 °C, 700–900 rpm, pH = 6.0 in 96 h	89.84 ± 0.41	2.80 ± 0.02	0.94 ± 0.00	2.62 ± 0.01	This study
*Rhodotorula paludigena* CM33	Glucose (batch phase), Sucrose (fed-batch phase)	Stirred tank bioreactor at 30 °C, 700–900 rpm, pH = 6.0 in 96 h	87.78 ± 0.30	3.25 ± 0.03	0.91 ± 0.02	2.97 ± 0.02	This study

The study further emphasized the natural capabilities and efficiency of *R. paludigena* CM33, a strain isolated directly from nature^8^. Its ability to thrive under various conditions and efficiently utilize sucrose as a substrate not only enhanced cost-effectiveness but also reduced overall cultivation time^53^. These attributes make sucrose-based media particularly promising for future development in fermentation processes, especially for applications in the animal feed, food, and pharmaceutical industries^54^. The high yields of protein biomass and β-carotene associated with this strain highlight its potential for commercial-scale applications.

### Nutritional composition

To investigate their feasibility for commercial development in animal feed supplement, dried *R. paludigena* CM33 cells were nutritionally analyzed after fed-batch cultivation in a 22L-bioreactor. Table 4 offers a comprehensive view of the nutritional composition of *R. paludigena* CM33 under various cultivation techniques and mediums, which is critical for evaluating its potential for commercial development in animal feed supplement. The data reveals several essential insights regarding the yeast's nutritional profile. The analysis of key compounds, carotenoids accumulation, conformed the potential health-promoting and biotherapeutic properties of *R. paludigena* CM33. Directly, accumulation of carotenoids in the cells possibly makes *R. paludigena* CM33 as a colorant for promoting pigmentation in ornamental and consumed aquatic animals. Additinally, this indicates its potential to enhance the growth, disease resistance, and meat quality of aquatic animals when used as a probiotic supplement in their diet, as demonstrated by Sriphuttha et al.^5^.

**Table 4:** Comparative analysis of nutritional compositions of *R. paludigena* CM33 cultivation in different cultivation techniques and carbon sources.

Nutrients	RPO	RPG (means ± SD)	RPS (means ± SD)
Moisture (%)	2.22	2.88 ± 0.04	3.62 ± 0.11***
Crude protein (%)	40.69	42.96 ± 0.53	42.47 ± 1.19
Crude lipid (%)	4.54	2.60 ± 0.02	2.89 ± 0.12*
Crude fiber (%)	0.56	1.05 ± 0.08	1.42 ± 0.04
Ash (%)	9.51	9.48 ± 0.72	11.00 ± 0.68
Nitrogen-free extract (%)	42.48	41.03 ± 1.23	38.60 ± 1.19
Calcium (%)	0.62	0.37 ± 0.01	0.35 ± 0.02
Phosphorus (%)	1.00	1.24 ± 0.01	1.49 ± 0.03***
β-carotene (mg/100 g)	222.20	280.09 ± 2.33	325.14 ± 2.73***
References	Thumkasem et al.^4^	This study	This study

RPO = *R. paludigena* CM33 cell from batch fermentation under optimal conditions; RPG = *R. paludigena* CM33 cell from fed-batch fermentation using Feed Medium 3; and RPS = *R. paludigena* CM33 cell from fed-batch fermentation using Sucrose Feed Medium. The data from this study (RPG and RPS) are given as means ± SD (*n* = 3). Student *t*-test was used to compare nutrient data between RPG and RPS: P < 0.05 (*), P < 0.005 (**), and P < 0.001 (***).

Proteins constitute significant dietary needed for animal growth, and are the dominant organic material in animal tissue^55^. In contrast to carbohydrates (nitrogen-free extract), proteins constitute a major cost for aquafeed development due to it’s the costliest ingredient of dietary preparation^56^. In the present study, cultivating *R. paludigena* CM33 with sucrose feed demonstrated a negligible alteration in crude protein content (42.47%) compared to the glucose feed (42.96%), highlighting its suitability as a feed supplement for animals. Both variants, RPG and RPS, contain substantial protein content and essential nutrients necessary for animal feed utilization, positioning them as valuable resources in animal nutrition. For crude lipid, under optimized conditions, this strain is promising for lipid production as it accumulates lipids to > 20% of dry cell weight, and long chain fatty acids (C16 and C18) are predominant, especially oleic acid (C18:1) and palmitic acid (C16:0)^8^. However, fatty acid profile was not emphasized in the present study due to relatively low amounts of detected crude lipid contents. This is also for the effects from other nutritional components (crude fiber, ash, calcium, and phosphorus) since the inclusion level of supplement is relatively low in practical feed.

### In vitro digestibility of protein and carbohydrate

**Figure 2:**
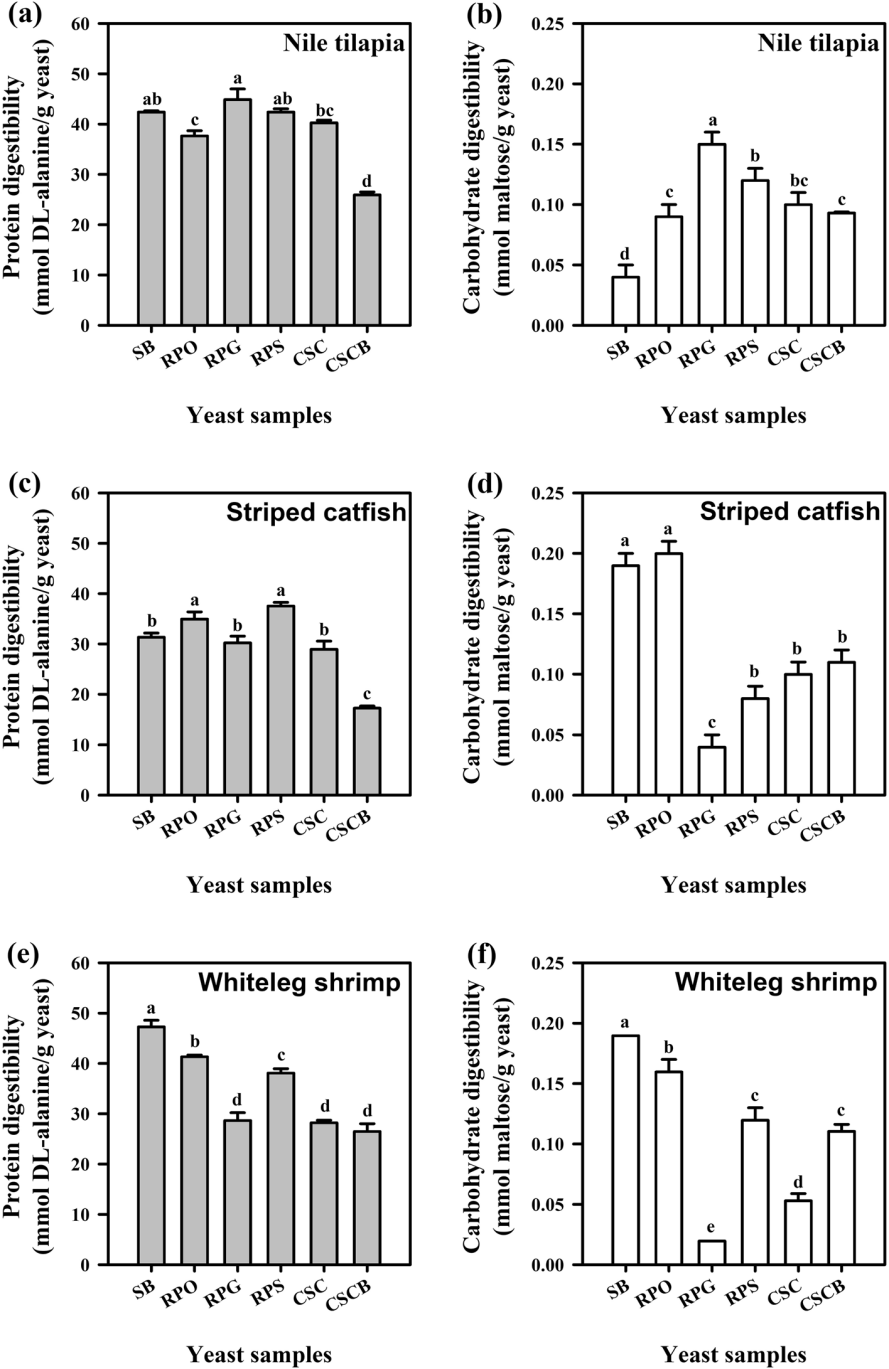
The in vitro digestibility of protein (mmol *DL*-alanine equivalent/g sample, gray bars) and carbohydrate (mmol maltose/g sample, white bars) in yeast samples using digestive enzyme extracted from Nile tilapia (**a**,**b**), striped catfish (**c**,**d**), and whiteleg shrimp (**e**,**f**). Data are expressed as means ± SD (*n* = 4). Significant differences between treatments are indicated by different superscripts (P < 0.05). SB = *S. bouladii*; RPO = *R. paludigena* CM33 cultivated in optimal conditions; RPG = *R. paludigena* CM33 cultivated in glucose feed; RPS = *R. paludigena* CM33 cultivated in sucrose feed; CSC = commercially available yeast *S. cerevisiae*; and CSCB = commercially available yeast *S. cerevisiae* blended with multi-strain probiotics.

Protein digestibility acts as a primary indicator in assessing feed ingredients for aquaculture purpose^57^. In general, yeast cells, such as *S. cerevisiae*, contained high amounts of essential nutrients, including proteins and carbohydrates^58^. As illustrated in Fig. 2, yeast samples and aquatic animal species had significant effects on IVDP and IVDC (P < 0.05). The commercially available yeast brands (CSC and CSCB) provided relatively low IVDP relative to SB or three alternative *R. paludigena* CM33 (RPO, PRG, and RPS) (P < 0.05). Using the digestive enzymes from Nile tilapia, better protein digestibility was observed in RPG and RPS samples relative to RPO. The different findings found in striped catfish and whiteleg shrimp indicate that RPO and RPS treatments achieved superior IVDP relative to RPG (P < 0.05). For IVDC, the yeast RPO was suitable for use as a feed supplement in three tested animal species. They provided moderate IVDC when screening by digestive enzymes from Nile tilapia but provided relatively high IVDC in the case of enzymes from striped catfish and whiteleg shrimp.

Since in vitro digestibility correlates with nutrient bioavailability^59–61^, our investigations suggest the potential of *R. paludigena* CM33 as an animal feed supplement, especially for aquatic animals. However, detailed composition, such as amino acid profiles, should be clarified before being used as an alternative feed supplement. Not only nutritive values but also yeast cells contain a number of active ingredients, which may promote the growth and health status of reared animals review by Mahdy et al.^62^. Before use as feed supplement, the level of *R. paludigena* CM33 in a practical feed, in comparison with a control unsupplemented feed, should be optimized. In vivo trials, observing growth, feed utilization, whole-body composition, and health status would be assessment criteria for clarifying aquaculture outcome.

## Conclusion

This study provided critical insights into the β-carotene production dynamics of *R. paludigena* CM33. It highlighted that Feed Medium 3, with a threefold increase in micronutrient concentration, yielded the highest β-carotene and biomass production. These experiments also showed that sucrose, used as a carbon source in a 22-L bioreactor, outperformed glucose in terms of biomass yields and TRY metrics of β-carotene production, establishing its potential for large-scale use. Furthermore, this work found *R. paludigena* CM33 to be a promising supplement for aquatic animal feed, offering competitive advantages in protein and carbohydrate digestibility. This research advanced the understanding of *R. paludigena* CM33’s production capabilities and emphasized the effectiveness of cost-efficient carbon sources, marking an important step in optimizing industrial bioprocesses for β-carotene production.

## Data Availability

The datasets used in this study, including any supporting raw data, are available upon reasonable request from the corresponding author.

## Abbreviations

CSC: Commercially sourced *Saccharomyces cerevisiae*
CSCB: Commercial blend containing *S. cerevisiae*
DOT: Dissolved oxygen tension
IVDC: In vitro digestibility of carbohydrate
IVDP: In vitro digestibility of protein
OD: Optical density
RPG: *R. paludigena* CM33 cell from fed-batch fermentation using Feed Medium 3
RPO: *R. paludigena* CM33 cell from batch fermentation under optimal conditions
RPS: *R. paludigena* CM33 cell from fed-batch fermentation using Sucrose Feed Medium.
SB: *Saccharomyces boulardii*
TRY: Titer, rate, and yield
YPD: Yeast extract, peptone, dextrose
*µ*: Specific growth rate (h^−^^1^)
*p*: β-Carotene product concentration (mg/L)
*Q*_*p*_: β-Carotene production rate (mg/L·h)
*Q*_*x*_: Biomass production rates (g/L·h)
*x*: Biomass concentration (g/L)
*Y*_*p/N*_: β-Carotene yield based on a nitrogen substrate (mg β-carotene/g nitrogen)
*Y*_*p/s*_: β-Carotene yield based on substrate consumed (mg β-carotene/g glucose or mg β-carotene/g glucose equivalent when sucrose was used as subatrate)
*Y*_*p/x*_: β-Carotene yield based on the biomass (mg β-carotene/g cells)
*Y*_*x/N*_: Biomass yield based on a nitrogen substrate (g cells/g nitrogen)
*Y*_*x/s*_: Biomass yield based on substrate consumed (g cell/g glucose or g cell/g glucose equivalent when sucrose was used as substrate.)
